# Early Life Socioeconomic Circumstance and Late Life Brain Hyperintensities – A Population Based Cohort Study

**DOI:** 10.1371/journal.pone.0088969

**Published:** 2014-02-18

**Authors:** Alison D. Murray, Christopher J. McNeil, Sima Salarirad, Lawrence J. Whalley, Roger T. Staff

**Affiliations:** 1 Aberdeen Biomedical Imaging Centre, University of Aberdeen, Aberdeen, United Kingdom; 2 Department of Nuclear Medicine, NHS Grampian, Aberdeen, United Kingdom; University Of São Paulo, Brazil

## Abstract

**Context:**

There have been many reports confirming the association between lower childhood socioeconomic circumstance and cardiovascular disease but evidence for links with cerebrovascular disease is contradictory. Hyperintensities on brain magnetic resonance imaging are associated with vascular risk factors, cognitive decline, dementia and death. However, the relationship between childhood socioeconomic circumstance and these lesions is unclear.

**Objective:**

To test the hypothesis that childhood socioeconomic circumstance is associated with late life hyperintensity burden and that neither adult socioeconomic circumstance nor change in socioeconomic circumstance during life influence this effect.

**Design:**

Cohort study

**Setting:**

Community

**Participants:**

227 community dwelling members of the 1936 Aberdeen Birth Cohort aged 68 years, who were free from dementia.

**Main Outcome Measures:**

Relationship between early life socioeconomic circumstance (paternal occupation) and abundance of late life brain hyperintensities.

**Results:**

We find significant negative correlations between childhood socioeconomic circumstance and white matter hyperintensities (ρ = −0.18, *P*<0.01), and periventricular hyperintensities (ρ = −0.15, *P*<0.05), between educational attainment and white matter hyperintensities (ρ = −0.15, *P*<0.05) and periventricular hyperintensities (ρ = −0.17, *P*<0.05), and between childhood intelligence and periventricular hyperintensities (ρ = −0.14, *P*<0.05). The relationship is strongest for childhood socioeconomic circumstance and regional white matter hyperintensities, where there is a step change in increased burden from paternal occupation grades equivalent to a shift from “white collar” to “blue collar” paternal occupation. Significant correlations were also found between hypertension and hyperintensity burden in all brain regions (ρ = 0.15–0.24, *P*<0.05). In models that include hypertension, the magnitude of the effect of childhood socioeconomic circumstance is similar to and independent from that of hypertension.

**Conclusions:**

Childhood socioeconomic circumstance predicts the burden of brain white matter hyperintensities aged 68 years. The mechanism underlying this effect is unknown, but may act through fetal and/or early life programming of cerebrovascular disease. Future work to understand this vulnerability will inform strategies to reduce dementia and stroke.

## Introduction

The concept that adult disease has its origins in early life is not new [1], [2]. Poverty has a negative effect on health and links between childhood poverty and coronary heart disease were established over three decades ago [3]. The association between lower childhood socioeconomic circumstance and cardiovascular disease is confirmed [4]. A proposed mechanism is accelerated post-natal growth in babies of low birth weight leading to increased cardiovascular disease and insulin resistance in later life. Such accelerated post-natal growth is observed in formula fed infants, rather than breast fed infants, and infant feeding practice is influenced by socioeconomic circumstance [5].

The evidence that childhood poverty is a risk factor for late-life cerebrovascular disease is contradictory. The Collaborative Study demonstrated an inverse relationship between childhood socioeconomic circumstance and stroke in men [6] and women [7], but others have found no relationship in men [8] or women [9]. The Collaborative Study found that haemorrhagic stroke, was associated with lower childhood socioeconomic circumstance [10]. However, recently the French 3Cs study, in normal old people, reported an 80% increased risk of ischaemic stroke in a higher income group, contrary to expectations [11].

Our group has previously demonstrated that lower childhood socioeconomic circumstance is related to smaller hippocampal volume more than 50 years later [12]. We and others have demonstrated an association between brain hyperintensities and cognitive decline [13], mild cognitive impairment, dementia, and death [14], [15]. It is clear that cerebrovascular disease plays some role in cognitive decline and dementia, and is responsible for additional cognitive burden to that associated with imaging measures of Alzheimer's neuropathology.

Here, we hypothesise that poorer socioeconomic circumstance in childhood is also associated with greater burden of magnetic resonance imaging detected brain hyperintensities in late mid-life. We further hypothesise that this relationship is not influenced by social mobility in adult life.

## Methods

### Ethics statement

This study received the approval of the Local Research Ethics Committee: Grampian Research Ethics Committee, Department of Public Health, Summerfield House, 2 Eday Road, Aberdeen, AB15 6RE, and informed written consent from the participants was obtained.

### Participants

The Scottish Mental Survey of 1947 (SMS47) tested the intelligence of almost all (>95%) 11 year-old children in Scotland on 4^th^ June 1947 [16]. Intelligence was measured using the Moray House Test No. 12. This is a valid test of verbal reasoning, numerical and spatial abilities and has a correlation co-efficient of 0.8 with the Stanford Binet intelligence quotient test. In 1999 with the approval of the Local Research Ethics Committee, and with informed consent from the participants, survivors of the SMS47 were recruited to a longitudinal study of health and cognitive ageing. This group is known as the Aberdeen Birth Cohort of 1936 (ABC36), and is described in more detail in Whalley et. al [17], a total of 506 people agreed to take part. From this sample, 319 participants were randomly invited to undergo brain MRI and 249 consented to do so, 243 participants were successfully scanned with complete image data suitable for analysis.

#### Socioeconomic circumstance assessment

Volunteers were asked the occupation of their father at the time they sat the Moray House Test aged 11 years in 1947. Paternal occupation was used as an estimate of childhood socioeconomic circumstance. Estimates of parental income were not available. Based on previous work [12] participant occupation was used as an estimate of adult socioeconomic circumstance and was recorded as the best ever grade achieved. For both paternal and participant occupation, occupational grade was recorded as coded by the UK's Office of Population Statistics classification. This classifies occupational status as follows: 1, managerial; 2, professional; 3, lesser professional; 4, secretarial; 5, skilled manual; 6, semi-skilled ii; 7, semi-skilled I; 8, unskilled (ii), and 9,unskilled (i). This occupational classification gives higher status occupations, often with more complex cognitive demands, a low score and places unskilled manual workers in the highest classification, with the transition between clerical and manual workers between 4 and 5 [18]. For clarity, and consistency with the educational scale, this scale was inverted so 1 = lowest status to 9 = highest status.

#### Education assessment

Education is recorded as best qualification attained and ranged from no qualification (score 1) to completion of a professional or higher degree (score 9). In detail, lack of qualifications was coded 1; lower leaving certificate coded 2; higher leaving certificate coded 3; Scottish vocational certificate coded 4; Ordinary levels/Standard grades coded 5; Highers/Advanced Levels were coded 6, undergraduate or postgraduate (Masters) qualifications were coded 7 and 8 respectively and those with a professional or higher degree were coded 9.

### Hypertension classification

Participants were classified as hypertensive based on a past history of hypertension or because a new diagnosis of hypertension was made during clinical assessment as part of the ABC36 study. Systolic and diastolic blood pressure were measured after the participant sat resting for five minutes in a warm and quiet room and the mean of three measurements was used for classification. In addition to 73 participants in ABC36 with previously diagnosed hypertension, six had untreated hypertension with systolic blood pressure of more than 150 mm Hg and diastolic blood pressure of more than 95 mm Hg; thus, 79 (34%) of the ABC36 MRI sample were defined as hypertensive. This definition of hypertension is based on the World Health Organisation criteria at time of brain imaging [19] and is strict compared with criteria of other organisations (e.g. ScotPHO) and thus defines a smaller proportion of the population as hypertensive.

### Brain imaging and image analysis

Brain MRI was carried out between 2003–2005 (when participants were aged ∼68y) using a 1.5T NVi system (General Electric, Milwaukee, Wi) using T2 axial (TR/TE 4900/81.4, slice thickness 5 mm, space 1.2 mm) and fluid attenuation inversion recovery axial (FLAIR) (TR/TE 9002/1.33, TI 2200, slice thickness 5 mm, space 1.2 mm). Complete T2 and FLAIR MRI data were available in 243. Inclusion criteria for MRI were availability of childhood intelligence scores and ability to give informed, written consent. Exclusion criteria were neurologic illness (such as Parkinson's disease, multiple sclerosis, or stroke resulting in loss of independence), dementia (defined as MMSE score of less than 24), and the usual contraindications to MRI. T2 and FLAIR images were examined and scored by an experienced observer (ADM) using the semi-quantitative Scheltens' scale [20], which attributes scores based on number and size of hyperintensities at different brain locations. We have previously used this scale with good to excellent intra- and inter-observer reproducibility [21]. Regional scores for white matter (WMH), grey matter (GMH), periventricular (PVH), and infratentorial hyperintensities (ITH) and total Scheltens' scores were entered into the analyses.

### Statistical analysis

Potential differences in educational attainment, childhood and adult socioeconomic circumstance and hypertension between cohort members who volunteered for MRI scans, and those who declined or were excluded were tested by one way ANOVA and chi squared test. Socioeconomic data are not normally distributed. Spearman's rho was used to identify relationships between variables. After dividing socioeconomic circumstance into high (≥6) and low (<6) groups, means and standard deviations of MRI and early life variables were examined. Finally, a structural equation model of the influence of childhood socioeconomic circumstance score on hyperintensity scores was constructed and the mediating effects of childhood intelligence, hypertension and education investigated.

## Results

### Descriptive statistics and correlations

**Figure 1:**
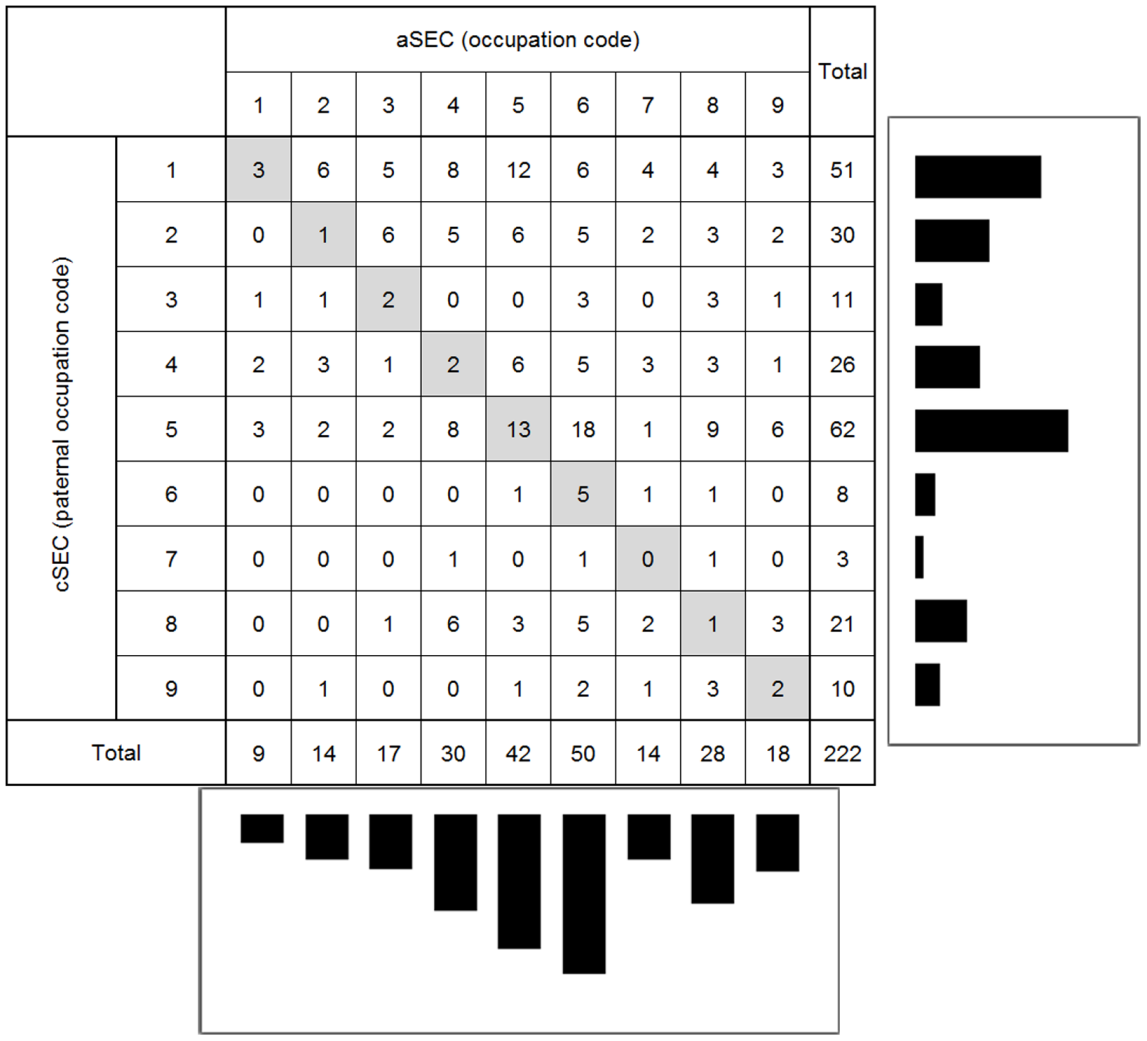
Childhood and adult socioeconomic circumstances compared. A comparison of the highest occupational status of the participant (adult SEC, aSEC) and that of their father when the participant was aged 11y (childhood SEC, cSEC). Population histograms demonstrate the distribution of SECs at these stages.

**Figure 2:**
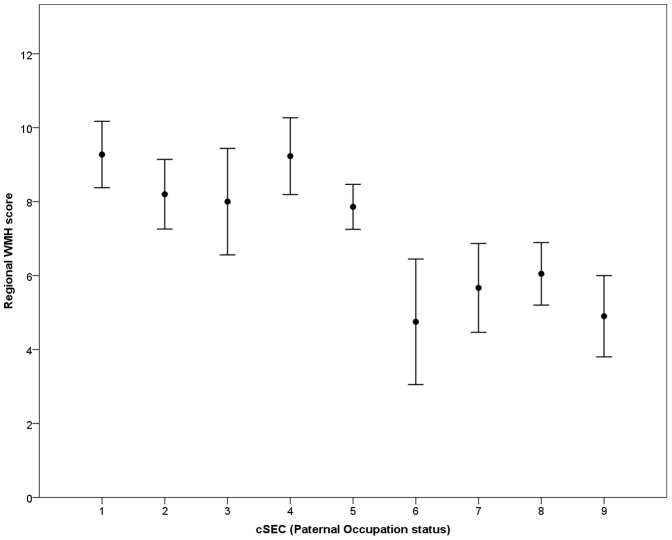
Brain regional WMH score versus paternal occupation category at participant age 11y. (+/− SEM, n = 222)

Participants in this brain imaging study had significantly higher cSEC (*P*<0.01), aSEC (*P*<0.05) and demonstrated a trend for better educational achievement than ABC36 participants who did not undergo MRI (Table 1). There was no difference in prevalence of hypertension or plasma levels of cholesterol and triglyceride between these groups. Virtually complete measures of childhood and adult socioeconomic circumstance, and education data were available in 227 participants (108 male) with MRI data. Seven of these participants had one missing data point. Median values for the group were used in these cases. Examination of paternal and participant occupation codes shows social mobility in both directions (Figure 1). The mean difference between paternal and participant occupational grade was 1.44, indicating a generational shift to occupations considered to be of higher social status. This reflects the known shift from “blue” to “white collar” occupations in the mid to late twentieth century. Individuals who experienced no social mobility were in the minority with only 13% unchanged since childhood. Inspection of the occupational score distributions indicated that they were not normally distributed in childhood or in adult life. Spearman's rho correlations between socioeconomic measures (cSEC, aSEC), childhood intelligence (CIQ), educational achievement (Edu), and MRI measures (WMH, PVH, GMH and ITH) are shown in Table 2. The results show no association between aSEC and hyperintensity burden. Education and cSEC were associated with PVH and WMH scores. Splitting the group into two (low and high) according to both paternal and own occupation (Table 3) at a SEC classification of >5 we found 42 participants had high cSEC and 115 had high aSEC. Eighty-seven moved from low cSEC to high aSEC and only 14 moved from high to low. Examining the MRI hyperintensity scores in both groups in terms of the other variables showed that the WMH scores were significantly higher in the low SEC groups (both childhood and adult) and PVH in the cSEC group. Education and childhood intelligence were significantly higher in the childhood and adult high SEC groups. These results indicate that early life environment and measures are associated with WMH and, to a lesser extent, PVH scores. These relationships are most obvious for regional WMH scores, where there is a cut-off between paternal occupation codes of 5 and 6 (Figure 2). It is unclear if these associations are independent of each other. Seventy-nine of the participants had a history of hypertension. Those with a history of hypertension had higher MRI scores for all regions (Table 3). There was no significant difference between those with a history of hypertension and those without in terms of education and childhood intelligence (Table 3). In addition using a chi squared test we found no association between aSES and cSES and a history of hypertension.

**Table 1:** Early life and adult socioeconomic circumstance, vascular risk factors and hypertension status of participating and non-participating members of ABC36 at age 64y.

	Non Participant		Participant		
	(n = 265–269)		(n = 231–232)		t-test
	Mean	SEM	Mean	SEM	*P*-value
aSEC (1–9)	4.02	0.14	4.47	0.14	0.023
cSEC (1–9)	2.48	0.13	3.05	0.16	0.007
EDU (1–9)	2.93	0.13	3.31	0.15	0.052
Body Mass Index	27.0	0.13	26.8	0.15	0.523
Plasma Triglyceride (mM)	1.88	0.28	1.84	0.26	0.678
Plasma Cholesterol (mM)	5.68	0.07	5.61	0.06	0.506
	yes	no	yes	no	*P*-value
Hypertension status	79	186	73	158	0.666#

#calculated by Pearson's Chi-squared test

Abbreviations: aSEC  =  adult socioeconomic circumstance, cSEC  =  childhood socioeconomic circumstance, Edu  =  educational score.

**Table 2:** Spearman's correlation co-efficients between socioeconomic circumstance, early life parameters, hypertension and regional MRI hyperintensity scores.

	WMH	PVH	GMH	ITH
aSEC	−0.067	−0.045	−0.019	−0.043
cSEC	−0.181**	−0.146*	−0.101	−0.013
EDU	−0.149*	−0.167*	−0.070	−0.105
CIQ	−0.129	−0.141*	−0.011	−0.046
Hyp	0.241***	0.193**	0.240***	0.149*

Abbreviations: aSEC  =  adult socioeconomic circumstance, cSEC  =  childhood socioeconomic circumstance, Edu  =  educational score, CIQ  =  age 11 intelligence, Hyp  =  treated or new hypertension, WMH  =  white matter hyperintensities, PVH  = periventricular hyperintensities, GMH  =  grey matter hyperintensities, ITH  =  infratentorial hyperintensities, ***  = *P*<0.001, **  = *P*<0.01, *  = *P*<0.05. N = 227

**Table 3:** Comparison of MRI and early life variables between high and low SEC groups.

	cSEC		aSEC		Hypertension status	
	High (n = 42)	Low (n = 185)	High (n = 115)	Low (n = 112)	No (n = 148)	Yes (n = 79)
WMH	5.50±3.81	8.51±5.43***	7.20±4.71	8.72±5.79*	6.97±4.99	9.64±5.50***
PVH	4.02±1.77	4.72±2.00*	4.32 ±1.99	4.81±2.00	4.24±1.97	5.12±1.91**
GMH	2.17±2.45	2.55±2.71	2.42±2.78	2.52±2.58	1.94±2.19	3.42±3.20***
ITH	1.19±1.61	1.56±2.53	1.38±2.32	1.62±2.47	1.15±1.81	2.16±3.14**
CIQ	104.8±15.2	98.9±14.6*	105.7±13.2	94.2±14.6***	100.9±15.3	98.2±14.4
EDU	3.95±2.29	3.08±2.17*	4.47±2.39	2.06±1.15***	3.35±2.15	3.01±2.29

aSEC  =  adult socioeconomic circumstance, cSEC  =  childhood socioeconomic circumstance, EDU  =  educational score, CIQ  =  childhood intelligence score, GMH  = grey matter hyperintensities, ITH  =  infratentorial hyperintensities, PVH  =  periventricular hyperintensities, WMH  =  white matter hyperintensities. Mean ± SD. *P<0.05, **P<0.01, ***P<0.001

### Structural equation modelling

**Figure 3:**
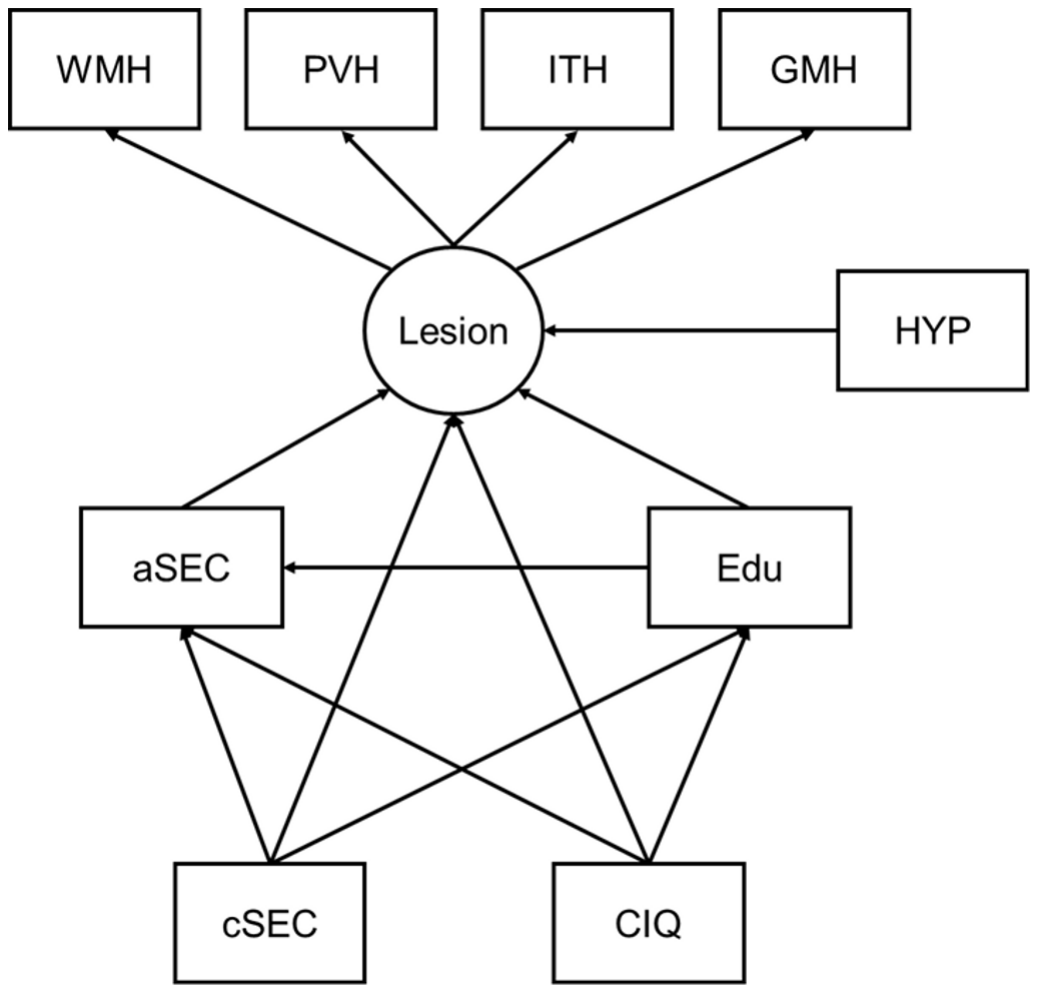
The relationship between childhood socioeconomic circumstance and late life whole brain hyperintensity burden. Structural equation model examining the relationship between childhood socioeconomic circumstance and late life whole brain hyperintensity burden, correcting for the mediating effects of childhood intelligence, education and adult socioeconomic circumstance. aSEC  =  adult socioeconomic circumstance, cSEC  =  childhood socioeconomic circumstance, Edu  =  educational score, CIQ  =  childhood IQ, lesion  =  latent variable contributed to by regional hyperintensity scores, GMH – grey matter hyperintensities, ITH  =  infratentorial hyperintensities, PVH  =  periventricular hyperintensities, WMH  =  white matter hyperintensities, HYP  =  treated or new hypertension

In order to examine the direct and indirect effects of cSEC on hyperintensity scores and to examine if this effect is a general effect rather than specific to WMH we used structural equation modelling. The model shown in Figure 3 hypothesises that all of the hyperintensity scores can be explained by a single ‘lesion’ latent variable. Childhood intelligence (CIQ) is hypothesised to have a direct effect on education (Edu), adult socioeconomic circumstance (aSEC), and lesion burden. Childhood socioeconomic circumstance (cSEC) is hypothesised to have a direct effect on lesion burden, aSEC and education. Hypertension was hypothesised to have a directed effect on lesion burden, as we have previously demonstrated [13]. Childhood SEC, hypertension, and CIQ were hypothesised to be correlated. A schematic representation of the model can be seen in Figure 3. For clarity, error terms, correlation arrows and regression weights have been excluded. Initial examination of the model using modification criteria of Joreskog and Sorbom [22] indicated that there was no modification that would substantially improve the fit of the model to the data. The regression weights for each hypothesized path can be seen in Table 4. The data were found to be an excellent fit to the model as indicated by the following indices; Chi Squared/df  = 1.31; Normed fit index  = 0.95 [23]; Comparative fit index  = 0.99 [24]; Root mean square error of approximation  = 0.037 [25]. The absence of any significant modification suggests that the effect of the hypothesised predictive variables is explained by their effect on the latent variable ‘lesion’ which estimates the shared variance between all regional hyperintensity scores rather than affecting one variable in particular. Calculating the direct and indirect influence of cSEC on this latent variable ‘lesion’ demonstrate that its influence on lesion burden is entirely direct.

**Table 4:** Regression weights for SEM illustrated in Figure 3.

			Std Beta	Beta	S.E	CR	*P*-value
cSEC	**→**	Edu	0.068	0.062	0.055	1.124	0.261
**CIQ**	**→**	**Edu**	0.449	0.091	0.012	7.456	<0.001
cSEC	**→**	aSEC	0.103	0.09	0.048	1.882	0.06
**CIQ**	**→**	**aSEC**	0.165	0.032	0.012	2.721	0.006
**Edu**	**→**	**aSEC**	0.482	0.458	0.058	7.968	<0.001
**Hyp**	**→**	**lesion**	0.295	0.964	0.239	4.03	<0.001
**cSEC**	**→**	**lesion**	−0.218	−0.141	0.047	−3.024	0.002
CIQ	**→**	lesion	−0.081	−0.012	0.011	−1.045	0.30
Edu	**→**	lesion	−0.112	−0.079	0.061	−1.294	0.192
aSEC	**→**	lesion	0.108	0.080	0.063	1.278	0.201
**lesion**	**→**	**GMH**	0.584	1			
**lesion**	**→**	**ITH**	0.526	0.806	0.123	6.532	<0.001
**lesion**	**→**	**PVH**	0.780	1.001	0.117	8.571	<0.001
**lesion**	**→**	**WMH**	0.900	3.071	0.35	8.762	<0.001

Beta  =  regression weight, std. Beta  =  standardized regression weight, S.E  =  standard error, CR  =  critical ratio, aSEC  =  adult socioeconomic circumstance, cSEC  =  childhood socioeconomic circumstance, Edu  =  educational score, CIQ  =  childhood IQ, lesion  =  latent variable contributed to by regional hyperintensity scores, GMH – grey matter hyperintensities, ITH  =  infratentorial hyperintensities, PVH  =  periventricular hyperintensities, WMH  =  white matter hyperintensities, Hyp  =  treated or new hypertension. Figure 3 summarises the structure of the model. Interactions in bold are statistically significant (p<0.05).

## Discussion

Here we demonstrate that lower socioeconomic circumstance in childhood (cSEC), estimated from paternal occupation when participants were around age 11, is associated with greater burden of brain MRI hyperintensities in the seventh decade of life. This association is most obviously seen in the relationship between cSEC and subcortical and deep white matter hyperintensities (WMH). This association is independent of hypertension during life. All of the hyperintensity measures demonstrate a significant association or trend and modelling of the common variance shared by these measures (‘lesion’) suggested that this is a common effect rather than specific to a particular hyperintensity measure.

Adult socioeconomic circumstance does not predict regional hyperintensities or the latent variable ‘lesion’. The magnitude of the effect of cSEC on brain hyperintensities is similar to and independent to that of hypertension. Thus, the negative effect of low cSEC on brain hyperintensities is in addition to the established effect of hypertension [13]. The numerous ways in which we have tested this relationship indicate that cSEC has a significant underlying direct effect with no evidence of mediation by childhood intelligence, education, hypertension or adult SEC.

### Socioeconomic circumstance and ill health in general

The Black Report of 1980 made the link between poverty and poor health explicit in an attempt to place health inequalities firmly on the UK political agenda [26]. The influence of lower lifelong socioeconomic status on all cause mortality has been recognised for some time [6]. In addition to absolute poverty, which has understandable adverse effects on health, it is recognised that social inequalities underlie many of the negative associations between socioeconomic circumstance and adverse outcomes, including morbidity and mortality [27], [28]. What are less clear are mechanisms and timing of effects of poverty and socioeconomic inequalities on poor health. The results presented here demonstrate that childhood experience is measurable in the seventh decade and that early adversity is not overcome by better adult socioeconomic circumstance. Using this data set we have also shown that low cSEC is also associated with a small hippocampal volume [12], a recognised biomarker of Alzheimer's pathology [29]. This result was also found to be independent of later life SEC. Taken together these results indicate that the influence of low cSES on dementia-related neuropathologies may act through more than one route.

### Life-course change in socioeconomic circumstance and risk of cardiovascular disease

The Stockholm Female Coronary Risk Study showed women who were socioeconomically disadvantaged in early life or late life were both at increased risk of ischaemic heart disease and that late life disadvantage was a greater risk. However, the risk was greatest for those disadvantaged in both early and late life [30]. These results are somewhat contrary to the findings here, that effects on an imaging marker of cerebrovascular disease are dominated by socioeconomic circumstance in childhood.

### Socioeconomic circumstance and stroke

As described above, the epidemiology of ischaemic and haemorrhagic stroke differ, with evidence of increased risk of haemorrhagic stroke in those of cSEC disadvantage [10]. An Argentinian study found a similar relationship between adult socioeconomic circumstance and ischaemic stroke outcome and found unemployment (as an indicator of poorer current socioeconomic circumstance) to be a stronger determinant of in-hospital mortality than stroke severity or infarct size [31]. However, contrary to the study presented here the French 3Cs study, has reported an 80% increased risk of ischaemic stroke in a higher income group of a large population of community based elderly participants [11]. While brain hyperintensities are a risk factor, rather than a determinant of stroke, these findings are most likely to predict increased incidence of stroke in those of lower cSEC.

### Socioeconomic circumstance and white matter hyperintensities

There are few reports specifically investigating the relationship between brain MRI detected hyperintensities and socioeconomic circumstance but inference can be made from published data from one large population study. In the Northern Manhattan Study, which reported greatest WMH volumes in black participants, socioeconomic status was dichotomised as receiving Medicaid or not and had no significant influence on WMH [32]. However, two other large population based studies of WMH (LADIS and CASCADE), either do not capture socioeconomic circumstance or correct for measures from which this might be estimated [33], [34]. Nutritional factors associated with WMH include low vitamin B12 status, which itself is associated with elevated homocysteine and poor diet [35]. A further mechanism by which early life socioeconomic disadvantage influences vascular disease in general and brain imaging biomarkers of cerebrovascular disease in particular, is intrauterine growth [36]. It is hypothesised that growth acceleration in the early post-natal period or “catch-up growth” predicts the metabolic syndrome and that slower growth is favoured by breast feeding [37]. Individuals born very pre-term have altered white matter structure on diffusion tensor imaging as adults [38], low placental weight is associated with reduced white matter integrity in late life [39]. A socioeconomic gradient in white matter tract integrity has been demonstrated, partly mediated by obesity, smoking, and C-reactive protein, suggesting inflammation as a key mediating pathway [40]. Corticostriatal connectivity, which underlies reward signalling in response to positive feedback, is reduced in adults whose parents had less education [40], [41]. Thus suboptimal white matter development and increased disease burden in later life are both predicted by early life socioeconomic disadvantage.

### Strengths, weaknesses

The strengths of this analysis are the availability of estimates of life-long socioeconomic circumstance measures in well-characterised normal people, for whom structural brain MRI data exist. Weaknesses are lack of estimates of physical health earlier in childhood (or in utero) and a broader range of potentially confounding health measures during life. In addition, use of an individual's recollection of paternal occupation as the only measure of cSEC can be criticised because it does not capture the full extent of the socioeconomic circumstance and may be biased. However, the direction of bias of recollected paternal occupation is likely to underestimate the relationships demonstrated here.

### Conclusions and consequences for dementia risk

This analysis confirms that early life socioeconomic disadvantage is associated with increased brain imaging evidence of cerebrovascular disease in late midlife, with its established negative consequences for cognition, stroke, dementia and survival. Together with our previous report demonstrating smaller hippocampal volumes in those of poorer childhood socioeconomic circumstances [12], these results indicate that childhood disadvantage poses a “double whammy” of brain burden and provide insights into the disparity in dementia prevalence in those from different socioeconomic backgrounds [42]. The population affected is also less likely to benefit from the positive effects of prolonged education, a proxy of cognitive reserve [43]. Indeed, cSEC maybe be the origin of that reserve and conclusions that education protects against age-related cognitive decline may more accurately indicate that education as a surrogate of cSEC, rather than an estimate of intellectual endeavour. The over-riding importance of early life socioeconomic circumstance, compared with the influence of adult socioeconomic circumstance, suggests that policies which address social inequalities during pregnancy and early life will have the greatest impact on adult brain health, but that their influence on dementia prevalence will not be apparent for many years. Future work should model the influence of early life socioeconomic circumstance on brain imaging biomarkers of disease burden, life-course cognitive change and dementia risk.
